# Ameliorative effect of *Tiron* against paraquat-induced cerebral and pulmonary injury in rats: involvement of ferroptosis, TLR4/NF-κB and Nrf2/HO-1 signaling pathways

**DOI:** 10.1007/s00210-026-05033-1

**Published:** 2026-02-18

**Authors:** Nourhane M. Elemam, Manar A. Nader, Marwa E. Abdelmageed

**Affiliations:** 1 Department of Pharmacology and Toxicology, Faculty of Pharmacy, Mansoura University, Mansoura, 35516 Egypt; 2 Department of Pharmacology and Toxicology, Faculty of Pharmacy, Mansoura National University, Gamasa, 7731168 Egypt

**Keywords:** Paraquat, *Tiron*, TLR4/NF-κB, Nrf2/HO-1, Ferroptosis, Bronchoalveolar lavage fluid

## Abstract

Paraquat (PQ) is a herbicide which is used indiscriminately specially in developing countries. The objective of our study is to evaluate the toxicity of PQ on cerebral and pulmonary tissues and the efficacy of *Tiron* in attenuating such toxicity *through focusing in the involvement of Nrf2/HO-1 and TLR4/NF-κB signaling pathways*. Rats were divided into 5 groups of 6 rats each: control, *Tiron* control (200 mg/kg)*,* PQ (10 mg/kg), *Tiron* (100 mg/kg) + PQ, and *Tiron* (200 mg/kg) + PQ groups. Behavioural tests were performed before euthanasia. Subsequently, serum, brain, and lung samples were collected. Bronchoalveolar lavage (BAL) fluid and cell counting, serum iron and ferritin, oxidative stress biomarkers, histopathological examination along with immunohistochemical assessment of nuclear factor kappa B p65 subunit (NF-κB p65), nuclear factor erythroid 2**-**related factor 2 (Nrf2), and heme-oxygenase1(HO-1) were performed. Toll—like receptor 4 (TLR4), and interleukin 1beta (IL-1β) were also assessed. *Tiron* reversed PQ induced cerebral and pulmonary injury by enhancing motor activity confirmed by wire hanging and open field test (OFT), additionally, it decreased serum iron, total leucocytes count (TLC), monocytes and neutrophils count, 4-Hydroxynonenal (4-HNE), malondialdehyde (MDA), along with increased levels of serum ferritin, reduced glutathione (GSH), glutathione peroxidase 4 (GPX4), Nrf2, and total antioxidant capacity (TAC) in cerebral and pulmonary tissues beside improved histopathological alterations compared to PQ- administered group. Also, HO-1 was significantly increased along with a downregulation of TLR4, NF-κB p65, and IL-1β upon *Tiron* administration. *Tiron* mitigated PQ induced cerebral and pulmonary toxicity via restoration of ferroptosis balance, decreasing oxidative stress, and downregulation of inflammatory TLR4/ NF-κB pathway in a dose dependent manner.

**Graphical Abstract:**

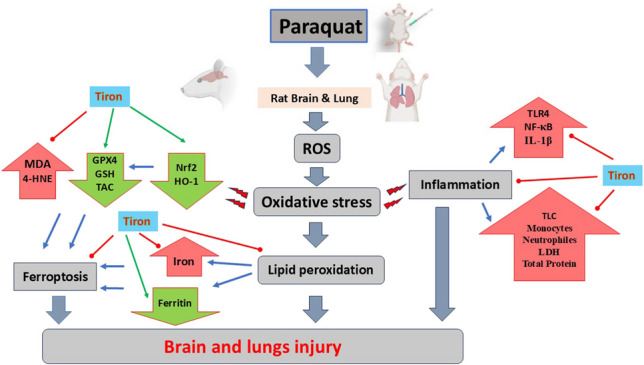

## Introduction

Paraquat (PQ) is a highly toxic bipyridyl herbicide that is widely used in agriculture to control weeds (Kermani et al. 2018). In various countries, PQ offers benefits to agriculture, but its toxicity poses significant concerns because PQ does not degrade quickly in the environment, and prolonged exposure can result in harmful bioaccumulation in humans and mammals, and due to the widespread use of PQ, it leads to residues accumulating on soil surfaces and in aquatic environments, ultimately entering the food chain (Huang et al. 2019). Surprisingly, PQ poisoning accounts for the highest number of fatalities, with a mortality rate ranging from 60 to 80% (Alizadeh et al. 2022). Unfortunately, there is no specific antidote for PQ intoxication (Zhang et al. 2019). PQ’s high toxicity, even in minute amounts, can cause rapid damage to more than one organ (Asaduzzaman et al. 2023). As a strong inducer of oxidative damage, PQ’s harmful effects on cerebral tissue were widely studied by scientists, as it was documented that PQ is a strong inducer of parkinsonism, behavioural, and memory changes in rodents (Lal et al. 2024; Beigoli et al. 2024). Additionally, previous experimental designs in rodents revealed that PQ is a strong inducer of severe lung injury that may lead to damage to alveolar epithelial cells, leading to pulmonary fibrosis, which is considered the main cause of death related to PQ intoxication (Shao and Chen 2014; Zheng et al. 2021).

PQ has been shown to be one of the strongest inducers of oxidative damage and inflammation that can affect nuclear factor erythroid 2**-**related factor 2 **/** heme-oxygenase 1 (Nrf2/HO-1) and toll—like receptor 4/nuclear factor kappa B (TLR4/NF-κB) pathways which are the major regulators of the activation of oxidative stress and inflammation (Akhigbe et al. 2024). Generally, Nrf2 is a basic leucine zipper transcription factor which is usually found sequestered to Kelch-like ECH-associated protein (Keap1) in normal conditions, and in case of oxidative stress it is released from Keap1 and then translocated into the nucleus for the transcription of Nrf2 gene, where it binds to antioxidant response element (ARE) to initiate the transcription of antioxidant enzyme HO-1 which is upregulated to promote cellular recovery, as it is reported that HO-1 and its metabolites have documented antioxidant anti-inflammatory activities (Yang et al. 2023; Shu et al. 2024; Liu et al. 2024). Moreover, TLR4/NF-κB pathway is usually activated due to the imbalance in oxidant/antioxidant status leading to the activation of TLR4 which is a cell surface receptor that is activated by several endogenous and exogenous ligands and upon its activation a signalling pathway involving NF-κB is initiated followed by activation of proinflammatory mediators, interleukin 1beta (IL-1β), interleukin 6 (IL-6), and tumour necrosis factor α (TNFα) (Akhigbe et al. 2024). Thus, the control of both pathways is postulated to offer a viable and efficient treatment of PQ induced cerebral and pulmonary injury.

Ferroptosis is a unique iron-dependent type of cell death that’s totally different from autophagy, necrosis, and apoptosis and mainly entails the fatal build-up of reactive oxygen species (ROS) and byproducts of lipid peroxidation as a result of iron metabolism (Xie et al. 2016; Stockwell et al. 2017). Excessive accumulation of iron leads to intense buildup of ROS via a Fenton reaction (Xie et al. 2016). Additionally, ROS can react with the polyunsaturated fatty acids in cellular membranes, leading to lipid peroxidation. Besides, nicotinamide adenine dinucleotide phosphate (NADPH)-dependent lipid peroxidation and the depletion in reduced glutathione (GSH) levels also play crucial roles in the induction of ferroptosis, where depletion of GSH leads to the inactivation of glutathione peroxidase 4 (GPX4), which is considered the direct regulator of ferroptosis (Xie et al. 2016). Studies have documented that ferroptosis can be involved in PQ toxic effects on cerebral and pulmonary tissues due to the intense imbalance in oxidant/antioxidant status causing severe neuroinflammation and lung fibrosis (Hou et al. 2019; Du et al. 2024).

*Tiron* (C_6_H_4_Na_2_O_8_S_2)_ is a potent antioxidant that counters the pathological effects of ROS production due to oxidative stress in various cell types (El Mahdy et al. 2024). As a potent superoxide scavenger, it was reported that *Tiron* has a dramatic protective effects on aluminium induced brain injury in rats, oxidative stress induced pulmonary injury in rats, nicotine-induced lung and liver injury in rats, as well as, 1-methyl-4-phenyl-1,2,3,6-tetrahydropyridine (MPTP) induced parkinsonism in mice (Sharma et al. 2007; Davidovich et al. 2013; Khaled et al. 2020; Mohamed et al. 2021). Also, *Tiron* was effective in modulating acetaminophen induced liver injury via mitigation of oxidative stress and inflammation (Shoeib et al. 2016).

Given the established antioxidant and anti-inflammatory properties of *Tiron*, we hypothesize that *Tiron* protects against paraquat (PQ)-induced brain and lung injury by activating the Nrf2/HO-1 antioxidant signaling pathway while concurrently inhibiting the TLR4/NF-κB inflammatory signaling pathway. Through this dual modulation, *Tiron* is expected to reduce oxidative stress, limit pro-inflammatory mediator release, and mitigate PQ-induced neurotoxicity and pulmonary injury.

## Material and methods

### Materials

PQ (dissolved in distilled water) was purchased from Tokyo chemical industry, Japan Cat No (C1749). *Tiron* (dissolved in normal saline (0.9% NaCl) was purchased from Sigma Aldrich Chemical Company (St. Louis, MO, USA, Cat No. 270573–71-2). Thiobarbituric acid (TBA) (Cat No. 504–17-6), Ellman’s reagent (Cat No. 69–78-3), trichloroacetic acid (TCA) (Cat No. 76–03–9), and sodium dodecyl sulphate (SDS) (Cat No. 151–21-3) were also purchased from Sigma Aldrich Chemical Company (St. Louis, MO, USA).

### Experimental animals

Male albino *Wistar* rats (200 ± 20 g weight) were purchased from “Egyptian Organization for Biological Products and Vaccines, Vacsera CO, Giza, Egypt”. Rats were observed for one week before starting the experimental study for adaptation. They were kept in the animal house of Faculty of Pharmacy, Mansoura University under standard nutritional and environmental conditions during the experimental period. The experiments for this study were conducted following the guidelines outlined in the National Institutes of Health Guide for the Care and Use of Laboratory Animals (NIH publication no. 85–23, revised 2011). The experimental design has been approved by Mansoura university animal care and use committee MU-ACUC, code number: (PHARM.MS.23.04.10).

### Experimental protocol

The rats (*n* = 30) were randomly divided into five groups; each group involve six rats as follows:

- **Group 1** (Normal control group): received intraperitoneal (i.p.) injection of normal saline daily for 14 days and i.p. injection of distilled water on day 7.
- **Group 2** (*Tiron* group): received *Tiron* (200 mg/kg) i.p. daily for 14 days (Khaled et al. 2020), *Tiron* was dissolved in normal saline (Khaled et al. 2020; El-Sherbeeny et al. 2016). A pilot study was conducted to assess the feasibility of chosen doses of *Tiron*.
- **Group 3** (PQ group): received i.p. injection of normal saline daily for 14 days and a single dose of PQ (10 mg/kg) dissolved in distilled water i.p. on day 7 (Chanyachukul et al. 2004; Ossowska et al. 2005).
- **Group 4** (*Tiron* 100 + PQ group): received *Tiron* (100 mg/kg) i.p. for 7 days, then a single i.p. dose of PQ (10 mg/kg) in day 7 followed by another 7 days of i.p. administered *Tiron* (100 mg/kg) (Khaled et al. 2020).
- **Group 5** (*Tiron* 200 + PQ group): received *Tiron* (200 mg/kg) i.p. for 7 days (Khaled et al. 2020) then a single i.p. dose of PQ (10 mg/kg) in day 7 followed by another 7 days of i.p. administered *Tiron* (200 mg/kg).

**Fig. 1:**
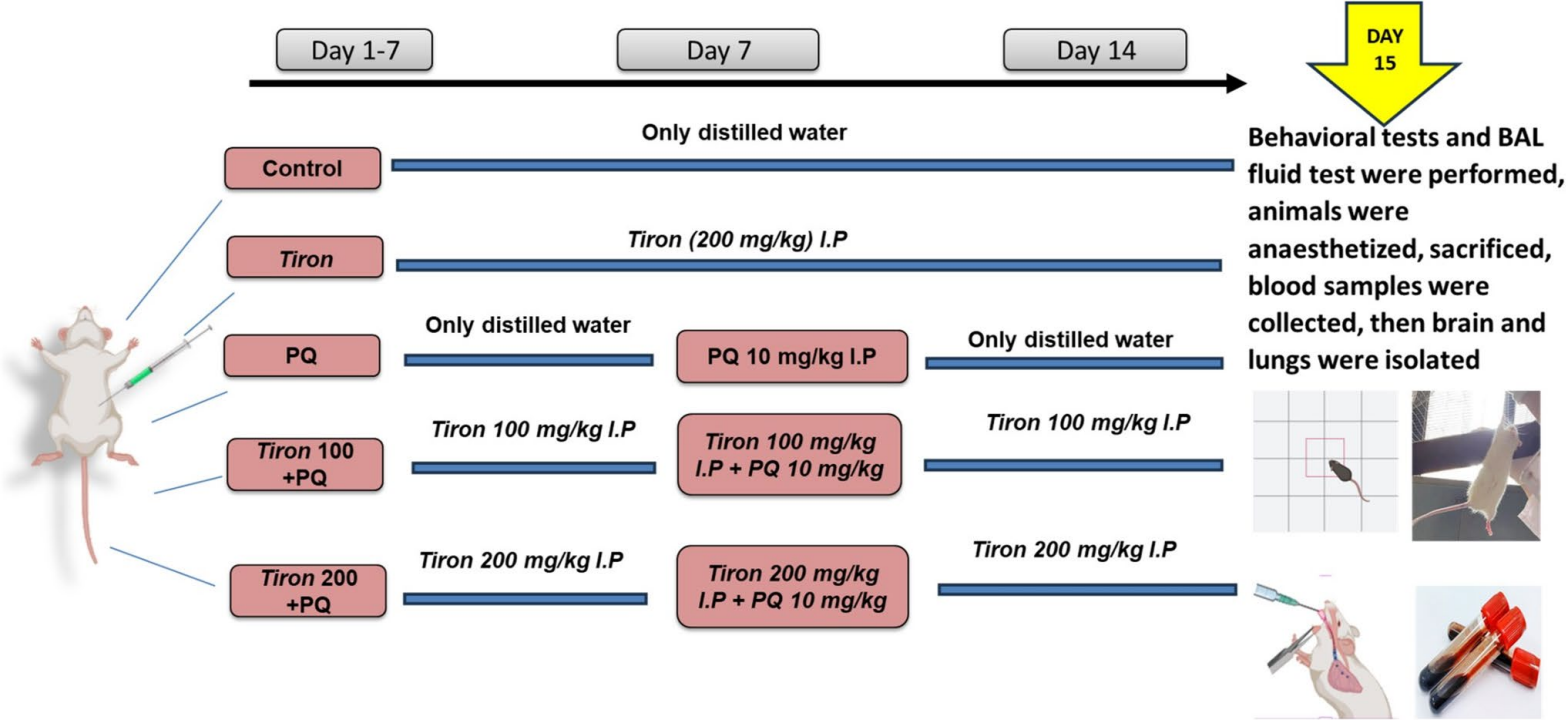
Schematic Diagram for conducted experimental design. PQ: paraquat, i.p.; intraperitoneal injection, BAL fluid; bronchoalveolar lavage fluid

Seven-days following PQ injection, 24 h after last *Tiron* dose, all rats were anesthetized using sodium secobarbital (40 mg/kg, i.p.) then samples were obtained (Fig. 1).

#### Behavioural tests

##### Wire hanging test

The experiment was done to assess the neuromuscular performance of the rats, and involved setting up a 50 cm steel wire horizontally (with a diameter of 2 cm), and positioned 40 cm above the floor. Rats were allowed to grasp the middle of the steel wire using their two forepaws for about 30 s, and the experiment was done in 3 different trials, then rats were scored according to their ability to maintain their grip and navigate on the wire over the 30 s of observation period as follows: 0: for rats that fell off the wire; 1: for rats hanging onto the steel wire with their forepaws; 2: for rats hanging onto the steel wire and attempting to climb; 3:for rats hanging onto the wire and one or both of their hind paws; 4: for rats hanging onto the wire with all four paws and their tails wrapped around the wire; 5: for rats that managed to escape to one of the platforms located at each end of the wire (Chen et al. 2024).

##### Open field test (OFT)

OFT test was performed to assess the rats’ spontaneous locomotor activity as mentioned in the previous study (Hall 1934). This test was performed in a quiet environment, and the rats were allowed to adapt to the laboratory for at least 10 min, then the rats were placed in the centre of wooden open field arena (100 × 100 cm^2^) and all movements of the rats were observed for 5 min, then the number of rearings and crossings was recorded.

#### Blood samples and tissue specimen collection

Blood samples were obtained from the retro-orbital venous plexus and were centrifugated at 672 g after allowing clotting for 15 min at 37 °C to obtain serum (Greenfield 2017). Resultant serum was frozen at − 80 °C till biochemical assay. Brain and lungs of all rats were removed and washed with normal saline (0.9%). Each tissue was subsequently weighed and divided as follow: for cerebral tissues: the right hemisphere was fixed in 10% buffered formalin for histopathological and immunohistochemical detection while, the left one was homogenized in phosphate-buffer saline (PBS), pH = 7.4, and frozen at −80 °C for measurement of different biomarkers. For pulmonary tissues, the right lobe was fixed in 10% buffered formalin for histopathological and immunohistochemical detection, while the left one was homogenized in PBS, pH = 7.4, and frozen at −80 °C for different measurements. For all rats, cerebral and pulmonary tissues were homogenized in PBS in an ice-cold beaker, then homogenates were centrifuged at (108 g/4^◦^C for 20 min) using a cooling centrifuge (Sigma D-37520, Germany). The resultant supernatant was divided into portions and kept at − 80 °C for further investigations. Another set of six animals in each group were preserved for bronchoalveolar lavage (BAL) fluid collection and analysis procedures.

### BAL fluid and cell counting

When the rats were anaesthetized, 6 ml of BAL fluid were gathered by lung perfusion via tracheal cannulation with saline. The resultant fluids were centrifuged for 10 min at 250 g at 4 °C using cooling centrifuge (Sigma D-37520, Germany) (Abdelmageed et al. 2016). The supernatants were aliquoted and used in the measurement of lactate dehydrogenase (LDH) and total protein content (TP), while the pellets were resuspended with 0.5 ml PBS and used to count total and differential leucocytes count using a hemocytometer (Mindray BC-3000 Plus, China) (Abdelmageed et al. 2016).

#### Determination of LDH in cerebral tissues

Cerebral level of LDH was determined following the manufacturer’s guidelines by commercially available kit from (Biomed Diagnostics, USA; Cat. No. LDH117090).

### Determination of histopathological changes in cerebral and pulmonary tissues

Cerebral and pulmonary tissues were kept in 10% formalin buffer, immersed in wax of paraffin, incised into sections, and stained with hematoxylin–eosin (H&E) dye and was examined using light microscope (Olympus CH2, Japan) (Layton and Suvarna 2013; Mirzaee et al. 2019; Palipoch et al. 2022). histopathological were recorded in 6 sections per slide and three photos were taken per section. The pathologist was blinded on control, diseased, and treated groups as samples are coded by groups and relevant background material including study design and objectives are disclosed to pathologist who worked independently and utilized a semiquantitative scale to assess pathological alterations. Scoring of cerebral lesions is as follows: 0 = no degenerative changes, no necrosis, and no inflammation; 1 = minimal degenerative changes, rare necrotic tissues, and infrequent rare inflamed cerebral tissues; 2 = moderate degenerative changes, minimal to mild necrotic tissues, and focal to multifocal inflammation; 3 = severe degenerative changes, moderate to severe necrosis, and dense coalescing inflammation.

Scoring of pulmonary alterations is as follows: 0 = no thickening of interalveolar septa, no bronchiolar damage, and no inflammation; 1 = minimal thickening of interalveolar septa, rare bronchiolar damage, and infrequent perivascular inflammatory aggregates; 2 = moderate thickening of interalveolar septa, minimal to mild bronchiolar damage, and frequent perivascular inflammation extending to alveolar septa with mild peribronchiolar inflammation; 3 = severe thickening of interalveolar septa, moderate to severe bronchiolar damage, and dense perivascular, peribronchiolar inflammation. The histopathological scores were recorded and analyzed and median ± interquartile range (IQR) of six sections was calculated and presented.

### Serum iron and ferritin

Serum iron and ferritin were assessed for all treatment group using the following kits: (cat number: JAI-CFE-005, Adipogen Life Sciences, San Diego, USA) and (cat number: 1107140, Spinereact, GIRONA, Spain) respectively.

### Determination of oxidative stress biomarkers in cerebral and pulmonary tissues

GSH, GPX4, total antioxidant capacity (TAC), malondialdehyde (MDA), and 4-Hydroxynonenal (4-HNE), were estimated in cerebral and pulmonary homogenates. GSH was estimated in cerebral and pulmonary tissues using a previously published technique (Ellman 1959). GPX4 and TAC were measured using the following commercially accessible kit: (cat number: CSB-EL009869RA, Cusabio, Houston, USA), and (cat number TA 2513, Biodiagnostics Co, Egypt) respectively. A method previously described by Ohkawa H et al. was used to assess MDA (Ohkawa et al. 1979). 4-HNE was measured using ELISA kit (cat number: RTFI01293, Assay Genie, Dublin, Ireland).

### Immunohistochemical assessment of NF-κB p65 and HO-1 in cerebral and pulmonary tissues

The immunohistochemical assessment of proinflammatory transcription factor NF-κB p65 and antioxidant HO-1 was performed using Avidin–Biotin Complex method (Guesdon et al. 1979) using polyclonal antibodies (cat number: A11201, ABclonal, Massachusetts, USA), and (cat number: sc-390991, Santa Cruz Biotechnology INC, Texas USA) respectively. The intensity of tissues’ staining was assessed by scoring, and the percentage of positively stained area was analysed using image j software analysis Fiji V2.9.0 (Fiji imagej.net, USA) across six sections per treatment.

### Determination of TLR4, IL-1β, and Nrf2 in cerebral and pulmonary tissues

ELISA kits were used to assess levels of TLR4 (cat number: SEA753Ra, Cloud-Clone Corp, USA), IL-1β (cat number: E0119Ra, Bioassay Technology Laboratory, China), and Nrf2 (cat number: RD-NFE2L2-Ra, Reddot Biotech, Canada).

### Statistical analysis

Data were presented as means ± standard deviation (S.D.). Normality of the data was tested using the Kolmogorov–Smirnov test. Statistical analysis and graphical representation were performed using Graphpad Prism V 8.0.1 (Graphpad Software Inc., San Diego, CA, USA). Parametric data were analyzed using regular one-way ANOVA followed by Tukey's post-hoc test for multiple comparison, while, non-parametric data, presented as median ± interquartile range (IQR), were analyzed using Kruskal–Wallis test followed by Dunn’s post-hoc test. Statistical significance was defined as *p* < 0.05.

## Results

### Wire hang test for assessment of motor function and OFT for assessment of locomotor activity

**Fig. 2:**
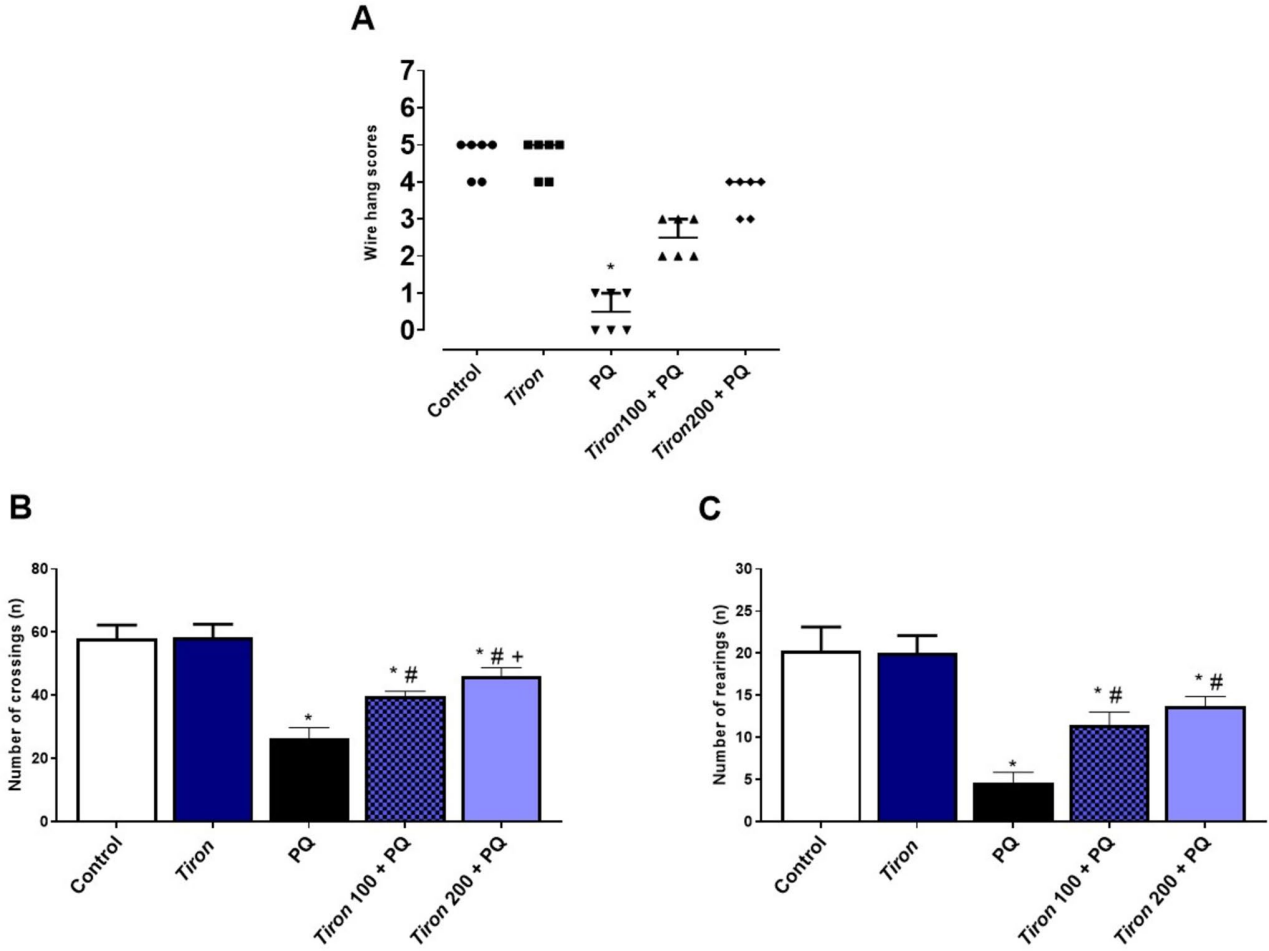
Effect of *Tiron* on wire hang test for assessment of motor function and OFT for assessment of locomotor activity. **A**: Wire hang test; **B**: Number of crossings; **C**: number of rearings. PQ; Paraquat, n; number, OFT; open field test. **A**: The data are presented as median ± IQR (*n* = 6). ^*^ *p* < 0.05, compared to the control group, Statistical analysis was conducted using Kruskal–Wallis test followed by Dunn’s multiple comparison test. **B**: Data are expressed as mean ± S.D. (*n* = 6). ^*, #, +^ *p* < 0.05 statistically significant difference vs. control, PQ, and (*Tiron* 100 + PQ) groups respectively (One-Way ANOVA followed by Tukey–Kramer multiple comparisons test)

The wire hang score showed that PQ rats had the shortest latency to fall among the other groups indicating that the motor function of rats was significantly impaired upon PQ intoxication. In contrast, (*Tiron* 100 + PQ) and (*Tiron* 200 + PQ) groups had longer latency to fall compared to PQ group indicating that *Tiron* could attenuate the motor dysfunction precipitated by PQ (Fig. 2A).

Additionally, OFT results showed a significant (*p* < 0.05) decrease in number of crossings in PQ group compared to control group evidencing impaired horizontal locomotor movements, while both *Tiron*- treated groups showed a markedly (*p* < 0.05) higher number of crossings compared to PQ group along with a higher number of crossings in (*Tiron* 200 + PQ) than in rats of (*Tiron* 100 + PQ) group, evidencing better locomotor function in (*Tiron* 200 + PQ) group (Fig. 2B).

Furthermore, a marked (*p *< 0.05) decrease in number of rearings was observed in PQ group compared to control group, while both groups of *Tiron* had a markedly (*p* < 0.05) higher numbers of rearings compared to PQ group which is consistent with the locomotor impairments described in number of crossings in PQ group along with a better performance seen in *Tiron* groups (Fig. 2C). *Tiron* group had insignificant difference from control group regarding performance in both behavioural tests.

### Effect of *Tiron* (100 and 200 mg/kg) on PQ-induced changes in BAL fluid cell count

**Fig. 3:**
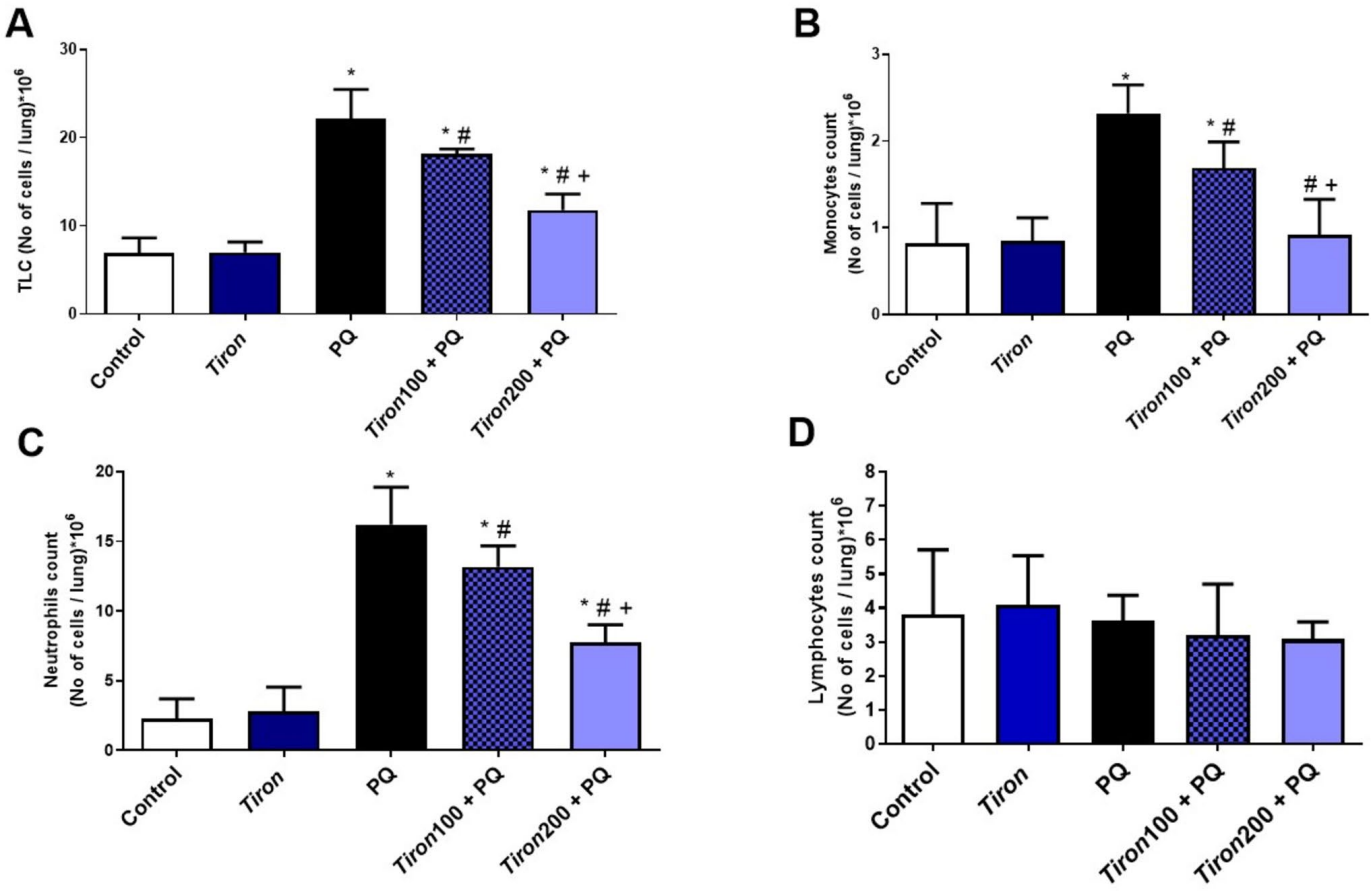
Effect of *Tiron* (100 and 200 mg/kg) on BAL cell count. **A**: TLC, **B**: monocytes count, **C**: neutrophiles count, **D**: Lymphocytes count. PQ; Paraquat, TLC; total leucocytes count, BAL; bronchoalveolar lavage. Data are expressed as mean ± S.D. (*n* = 6). ^*, #, +^ *p* < 0.05 statistically significant difference vs. control, PQ, and (*Tiron* 100 + PQ) groups respectively (One-Way ANOVA followed by Tukey–Kramer multiple comparisons test)

As shown in Fig. 3, PQ intoxication resulted in a marked (*p* < 0.05) increase in TLC, monocytes and neutrophils by 3.2, 2.8 and 7.0-folds (Fig. 3A, B, and C) respectively in comparison to control group. Whereas *Tiron* (100 and 200 mg/kg) showed a marked (*p* < 0.05) decrease in TLC by 17.96% and 46.86% respectively, a marked (*p* < 0.05) decrease in monocytes by 27.09% and 60.39% respectively, and a significant (*p* < 0.05) decrease in neutrophils by 18.70% and 52.10% respectively when compared with PQ group. These results showed a better effect of the dose (200 mg/kg) of *Tiron* in alleviating the massive increase in TLC, monocytes, and neutrophils induced by PQ compared to the lower dose of *Tiron.* Rats treated with *Tiron* (200 mg/kg) showed no significant difference from the control group but there was still a significant (*p* < 0.05) difference in TLC and neutrophiles count compared to control group. Similarly, Rats treated with *Tiron* (100 mg/kg) showed a significant (*p* < 0.05) difference regarding TLC, monocytes, and neutrophiles count compared to control group. *Tiron* group had insignificant difference from control group regarding prementioned markers.

Concerning lymphocytes, there was insignificant difference in lymphocytes count among all groups as described in Fig. 3D.

### Impact of *Tiron* (100 and 200 mg/kg) on LDH in brain tissue, LDH and TP content in BAL fluid

**Fig. 4:**
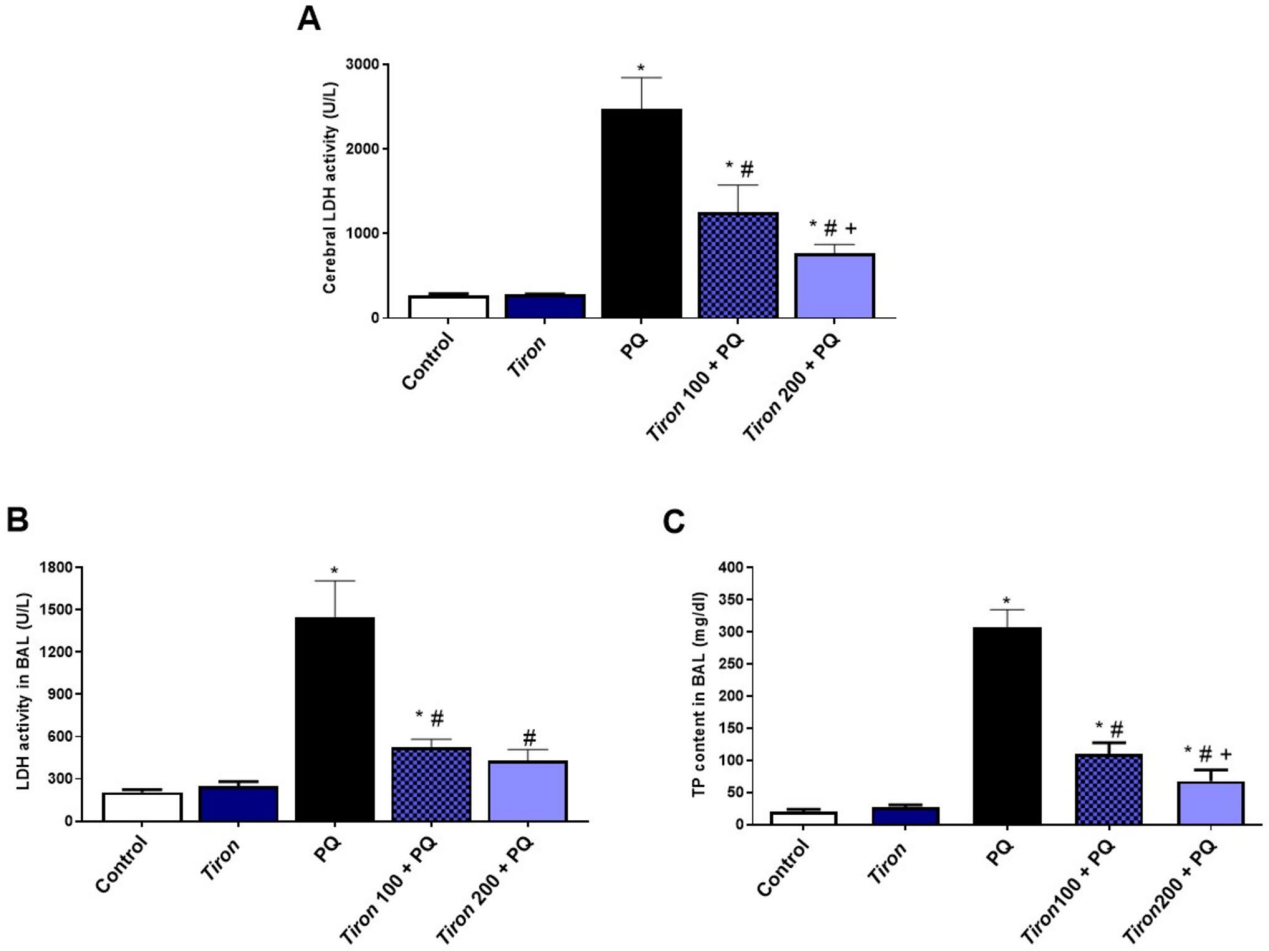
Effect of *Tiron* (100 and 200 mg/kg) on cerebral LDH activity and BAL fluid levels of LDH activity and TP content. **A**: Cerebral LDH activity; **B**: LDH activity in BAL fluid; **C**: TP content in BAL fluid. PQ; Paraquat, LDH; lactate dehydrogenase, TP; total protein, BAL; bronchoalveolar lavage. Data are expressed as mean ± S.D. (*n* = 6). ^*, #, +^ *p* < 0.05 statistically significant difference vs. control, PQ, and (*Tiron* 100 + PQ) groups respectively (One-Way ANOVA followed by Tukey–Kramer multiple comparisons test)

Toxicity with PQ resulted in a considerable (*p* < 0.05) increase in cerebral LDH activity (Fig. 4A), in addition, LDH activity in in BAL fluid was significantly increased by approximately 6.3-folds compared to control group (Fig. 4B), besides, a massive (*p* < 0.05) increase in TP content by 12.3-folds was marked (Fig. 4C). On the other hand, administration of *Tiron* (100 and 200 mg/kg) significantly decreased cerebral LDH activity and highly (*p* < 0.05) decreased LDH activity in BAL fluid by 59.80% and 65.97% respectively beside a massive (*p* < 0.05) decrease in TP content by 56.72% and 73.17% respectively compared to PQ group but both (*Tiron* 100 + PQ) and (*Tiron* 200 + PQ) groups showed a significant (*p* < 0.05) difference in TP content compared to control group, beside a significant (*p* < 0.05) difference in LDH activity in (*Tiron* 100 + PQ) group only compared to control group.

These results indicated a better effect of the higher dose of *Tiron* (200 mg/kg) in decreasing TP content in comparison with the lower dose, while both doses of *Tiron* had similar impact on LDH activity. *Tiron* group had insignificant difference from control group regarding both LDH activity and TP content.

### *Tiron* ameliorated histopathological alterations in cerebral and pulmonary tissues

#### Cerebral tissues

**Fig. 5:**
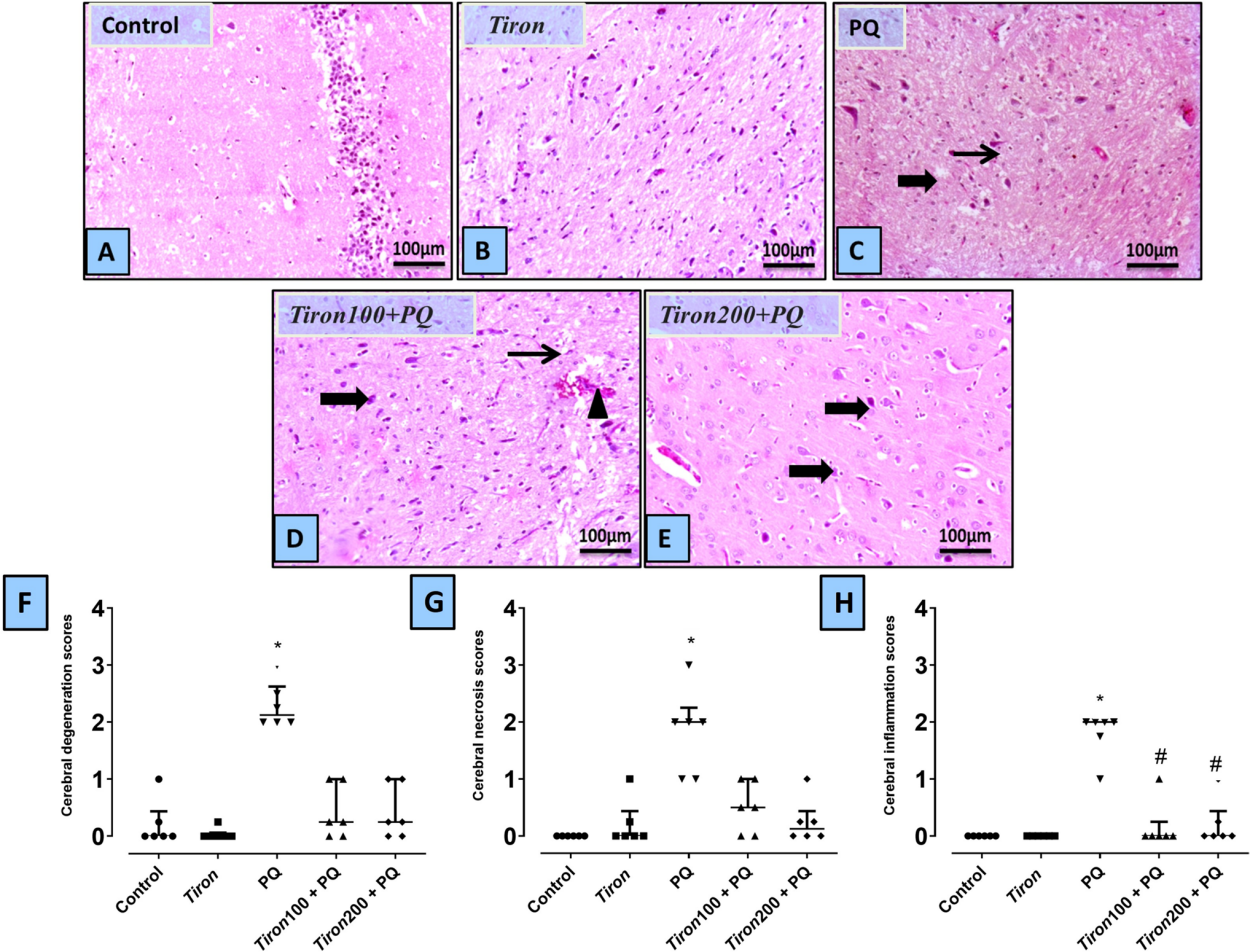
Effect of *Tiron* on histopathological alterations in cerebral tissues. **A**, **B**: sections of brain of control and *Tiron* groups (X: 100, bar = 100 µm); showed normal architecture of neuropil and neurological cells, **C**: sections of brain of PQ group (X: 100, bar = 100 µm, X 400, bar = 50 µm); showed neuronal cell necrosis (thin arrow) and diffuse neuropil vacuolation (thick arrow), **D**: sections of brain of (*Tiron 100* + PQ) group (X: 100, bar = 100 µm); showed few neuronal death (thick arrow), focal hemorrhage (arrowhead), moderate neuropil vacuolation (thin arrow), **E**: sections of brain of (*Tiron 200* + PQ) group (X: 100, bar = 100 µm); occasional neuronal death surrounded with few inflammatory cells (thick arrows). (**F**, **G**, **H**): scattered dot plot representing semi quantitative scoring of cerebral degeneration, necrosis, and inflammation respectively.. PQ; Paraquat. The data are presented as median ± IQR (*n* = 6). ^*, #^ *p* < 0.05 statistically significant difference vs. control, and PQ groups respectively. Statistical analysis was conducted using Kruskal–Wallis test followed by Dunn’s multiple comparison test

As clarified in Fig. 5, both control and *Tiron* control group showed normal architecture of neuropil and neurological cells (Fig. 5A and B). Contrarywise, PQ group showed neuronal cell necrosis and diffuse neuropil vacuolation (Fig. 5C). While (*Tiron* 100 + PQ) group showed few neuronal death, focal haemorrhage, moderate neuropil vacuolation (Fig. 5D), and (*Tiron* 200 + PQ) group showed occasional neuronal death surrounded with few inflammatory cells (Fig. 5E).

A semi quantitative assessment of cerebral degeneration (Fig. 5F), cerebral necrosis (Fig. 5G), and cerebral inflammation (Fig. 5H) showed an ameliorative effect on cerebral histopathological alterations in *Tiron* treated groups compared to PQ group.

#### Pulmonary tissues

**Fig. 6:**
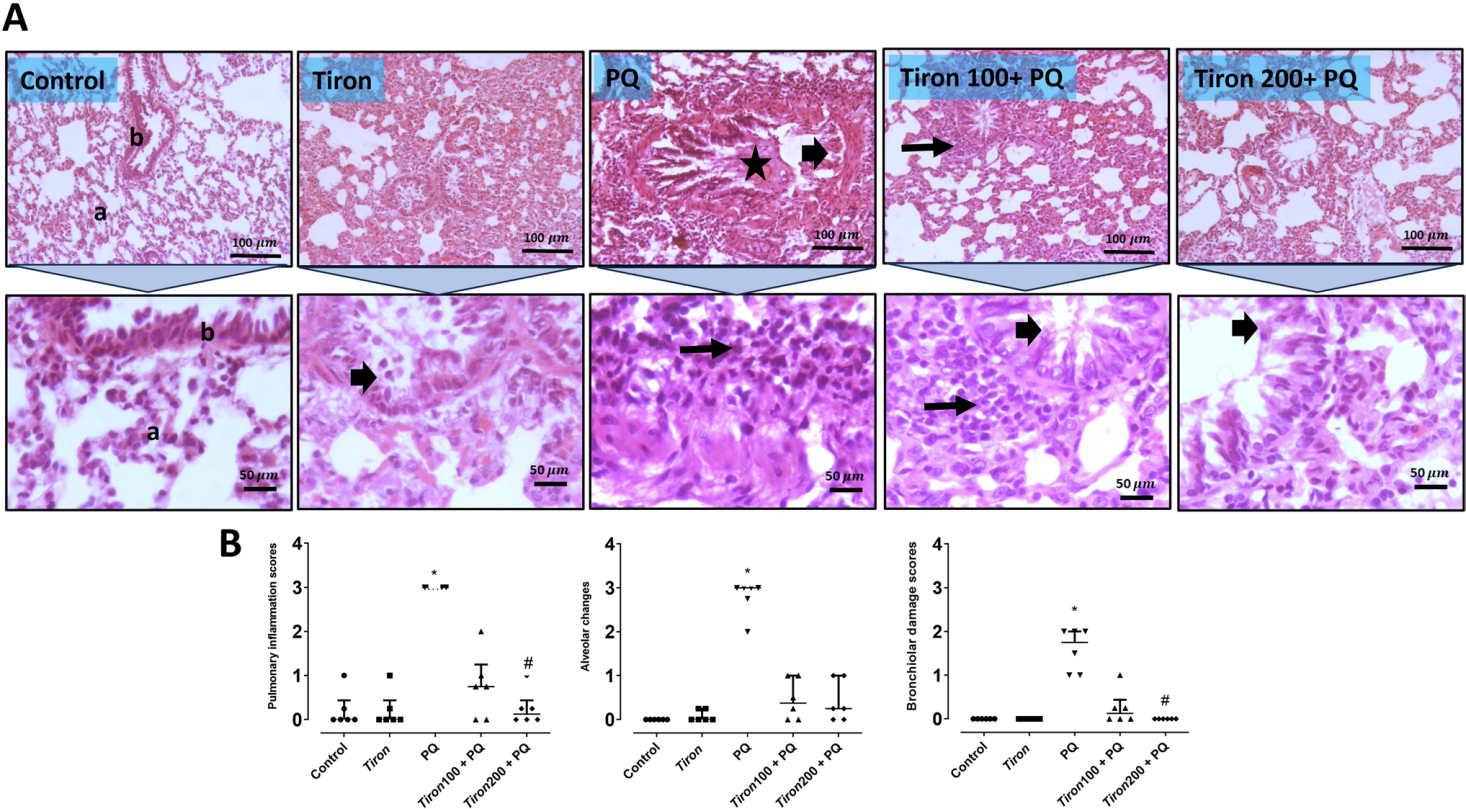
Effect of *Tiron* on histopathological alterations in pulmonary tissues. **A** Representative photomicrograph of pulmonary sections from different treatment groups (low and their corresponding high-power view). Control group showing normal histological appearance of bronchiolar and alveolar architecture. Trion showing approximately normal of the most architecture of pulmonary sections with occasional sloughing of bronchiolar epithelium. PQ group showing severe bronchiolar epithelial erosion and sloughing with intraluminal eosinophilic cellular debris and abundant peribronchiolar infiltrates including numerous lymphoplasmacytic cells, macrophages and neutrophils extending and expanding the interalveolar septa. *Tiron* 100 + PQ: showing focal mild to moderate peribronchiolar cellular infiltrates with focal bronchiolar epithelium sloughing forming intraluminal cellular debris. *Tiron* 200 + PQ: showing occasional bronchiolar sloughing with focal few peribronchiolar inflammation. A = intact alveoli, b = intact bronchiolar epithelium, Thin arrows = cellular infiltrates of inflammation, thick arrows = bronchiolar epithelial sloughing, arrowhead = alveolar atelectasis, stars = intraluminal cellular debris. Image magnification = 100x = Bar 100 μm, 400x = Bar 50 μm. **B** scattered dot plot representing semi quantitative scoring of pulmonary inflammation, alveolar changes, and bronchiolar damage respectively. PQ; Paraquat**.** The data are presented as median ± IQR (*n* = 6). ^*, #^ *p* < 0.05 statistically significant difference vs. control, and PQ groups respectively. Statistical analysis was conducted using Kruskal–Wallis test followed by Dunn’s multiple comparison test

**Fig. 7:**
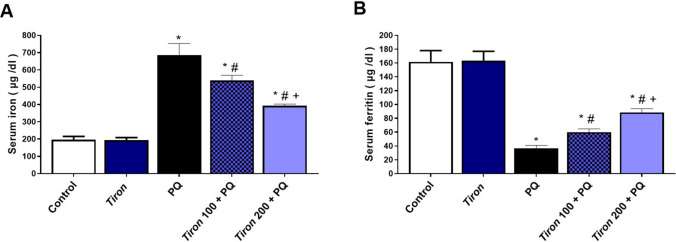
Effect of *Tiron* (100 and 200 mg/kg) on serum iron and ferritin levels. **A**: Serum iron, **B**: serum ferritin. Data are expressed as mean ± S.D. (*n* = 6). PQ; Paraquat. ^*, #, +^ *p* < 0.05 statistically significant difference vs. control, PQ, and (*Tiron* 100 + PQ) groups respectively (One-Way ANOVA followed by Tukey–Kramer multiple comparisons test)

As clearly shown in Fig. 6, both control group and *Tiron* control group showed normal architecture of pulmonary alveoli and bronchiolar structure, In contrast, Fig. 7C represents PQ group that showed severe bronchiolar epithelial erosion and sloughing with dense intraluminal eosinophilic cellular debris beside extensive peribronchiolar cellular infiltrates extending and expanding the interalveolar septa with alveolar atelectasis. Moreover, *Tiron* accomplished an ameliorative effect described in for (*Tiron* 100 + PQ) group which showed mild, focal to coalescing peribronchiolar aggregation of mononuclear cells together with few bronchiolar epithelial sloughing with formation of intraluminal cellular debris, and representing (*Tiron* 200 + PQ) group which surprisingly showed partial restoration of pulmonary architecture with approximately intact alveolar architecture, occasional bronchiolar sloughing and peribronchiolar inflammatory cells. A semi quantitative assessment of pulmonary inflammation, alveolar changes, and bronchiolar damage (Fig. 6B) showed a significant ameliorative effect on pulmonary histopathological alterations in *Tiron* treated groups compared to PQ group.

### Impact of *Tiron* (100 and 200 mg/kg) on serum iron and ferritin levels

PQ- injected group revealed a markedly (*p* < 0.05) elevated iron level by nearly 3.5-folds along with a significant (*p* < 0.05) decrease in ferritin level by 77.33% (Fig. 7A and B) which reflected intense build-up of iron within cerebral and pulmonary tissues. On the other hand, (*Tiron* 100 + PQ) group showed a marked decrease in serum iron by 21.52% along with an increase in ferritin level by 1.63-folds. Similarly, a dose of (200 mg/kg) of *Tiron* resulted in a substantial (*p* < 0.05) decrease in serum iron by 42.87% with an increase in serum ferritin by 2.41-folds indicating that the higher dose had better effect in the restoration of ferroptotic balance than (100 mg/kg) of *Tiron* but both groups still had a significant (*p* < 0.05) difference from control group*.* Additionally, *Tiron* group showed no significance from control group in both iron and ferritin levels.

### PQ intoxication alters oxidant/antioxidant balance related parameters in cerebral and pulmonary tissues

**Fig. 8:**
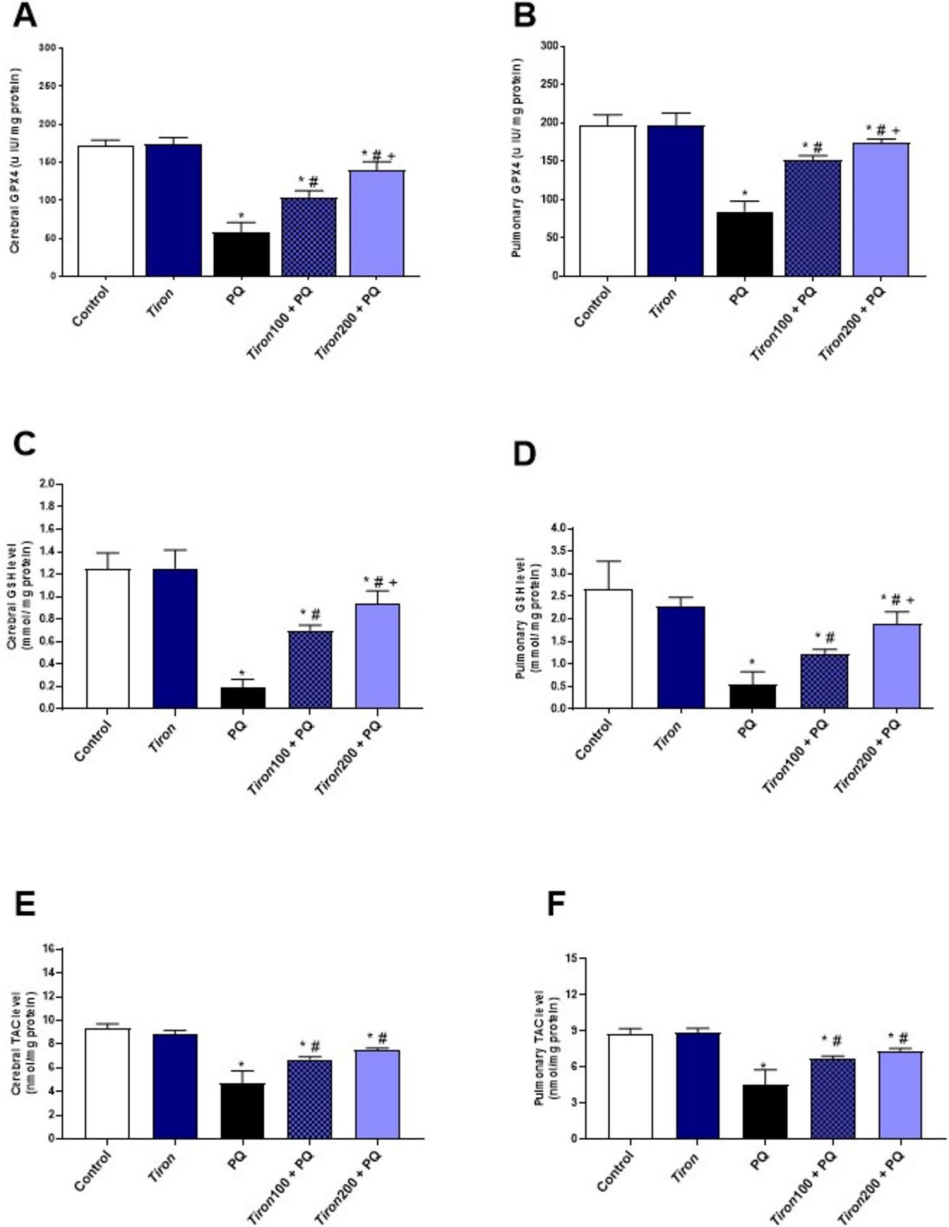
Effect of *Tiron* oxidant/antioxidant balance related parameters in cerebral and pulmonary tissues. **A**: Cerebral GPX4, **B**: pulmonary GPX4, **C**: cerebral GSH, **D**: pulmonary GSH, **E**: cerebral TAC, **F**: pulmonary TAC. PQ; Paraquat, GPX4; glutathione peroxidase 4, GSH; reduced glutathione, TAC; total antioxidant capacity. Data are expressed as mean ± S.D. (*n* = 6). ^*, #, +^ *p* < 0.05 statistically significant difference vs. control, PQ, and (*Tiron* 100 + PQ) groups respectively (One-Way ANOVA followed by Tukey–Kramer multiple comparisons test)

PQ administration resulted in a severe (*p* < 0.05) decrease in cerebral GPX4 and GSH in PQ group by 65.78% and 84.84% respectively (Fig. 8A and C) compared to control group, beside a decrease of the forementioned markers in pulmonary tissue by 56.92% and 79.26% respectively compared to control group as shown in Fig. 8B and D. Conversely, treatment with (100 mg/kg) of *Tiron* resulted in a considerable increase in cerebral GPX4 and GSH by 1.78 and 3.72 -folds beside a significant (*p* < 0.05) increase in pulmonary GPX4 and GSH by 1.79 and 2.22-folds respectively in comparison to PQ group. Similarly, treatment with (200 mg/kg) of *Tiron* resulted in a substantial increase in cerebral GPX4 and GSH by 2.38 and 4.96 -folds respectively, and in pulmonary tissues by 2.05 and 3.43-folds respectively compared to PQ group which indicate that (200 mg/kg) of *Tiron* had better antioxidant effect than (100 mg/kg). Both (*Tiron* 100 + PQ) and (*Tiron* 200 + PQ) groups showed a significant (*p* < 0.05) difference compared to control group. Additionally, *Tiron* group showed no significance from control group in GPX4 and GSH in cerebral and pulmonary tissues.

Additionally, TAC levels were extremely (*p* < 0.05) reduced in PQ group by 49.34% in cerebral tissues (Fig. 8E) and by 47.72% in pulmonary tissues (Fig. 8F) compared to control group. Contrarywise, treatment with (100 mg/kg) of *Tiron* resulted in a significant (*p* < 0.05) increase in cerebral TAC by 1.42-folds and pulmonary TAC by 1.43-folds compared to PQ group. Moreover, treatment with (200 mg/kg) of *Tiron* led to a substantial (*p* < 0.05) increase in cerebral TAC by 1.59-folds and in pulmonary TAC by 1.61-folds in comparison to PQ group but both (*Tiron* 100 + PQ) and (*Tiron* 200 + PQ) groups were still significant (*p* < 0.05) from control group. Moreover, *Tiron* group showed no significance from control group in both cerebral and pulmonary TAC levels.

**Fig. 9:**
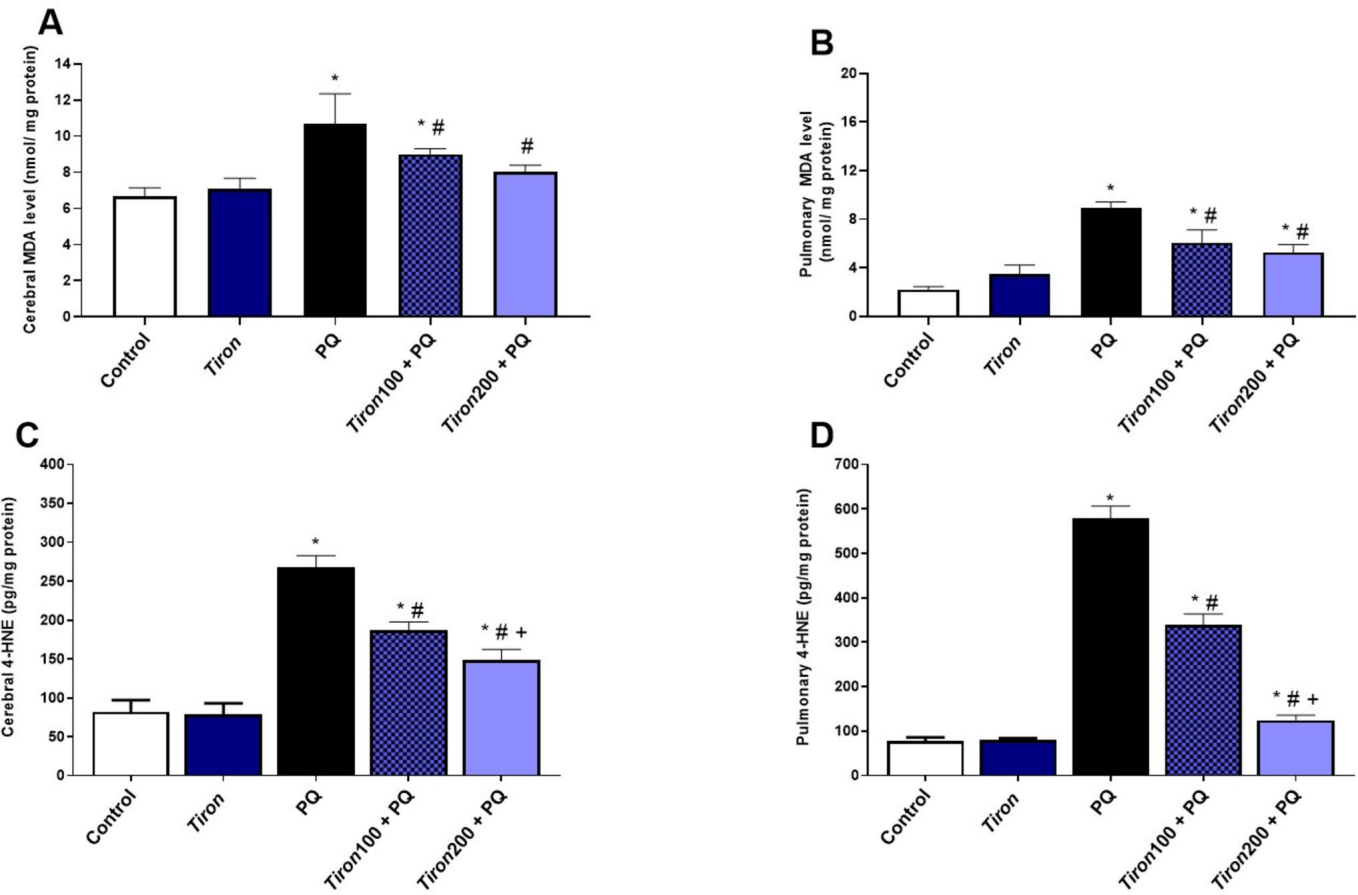
Effect of *Tiron* on tissue MDA and 4-HNE levels. **A**: Cerebral MDA, **B**: Pulmonary MDA, **C**: cerebral 4-HNE, **D**: pulmonary 4-HNE. PQ; Paraquat, MDA; malondialdehyde, 4-HNE; 4-Hydroxynonenal**.** Data are expressed as mean ± S.D. (n = 6). ^*, #, +^ *p* < 0.05 statistically significant difference vs. control, PQ, and (*Tiron* 100 + PQ) groups respectively (One-Way ANOVA followed by Tukey–Kramer multiple comparisons test)

As shown in Fig. 9, PQ intoxication led to a considerable (*p* < 0.05) increase in MDA and 4-HNE levels in cerebral tissues by 1.60 and 3.26-folds respectively (Fig. 9A and C), and in pulmonary tissues by nearly 4.06 and 7.45-folds respectively compared to control group (Fig. 9B and D) whereas, treated groups with (*Tiron* 100 + PQ) group decreased showed a significant (*p* < 0.05) reduction in cerebral MDA and 4-HNE levels by 15.57% and 30.06% respectively in cerebral tissues and 32.22% and 41.51% respectively in pulmonary tissues compared to PQ group. Additionally (*Tiron* 200 + PQ) group revealed a substantial (*p* < 0.05) decrease in cerebral MDA and 4-HNE levels by 24.64% and 44.51% respectively, and in pulmonary tissues MDA and 4-HNE were reduced by 40.95% and 78.49% respectively when compared to PQ group, findings that support that lipid peroxidation activity decreased in a dose dependent manner. (*Tiron* 100 + PQ) group was still significant from control group regarding cerebral and pulmonary MDA and 4-HNE, while (*Tiron* 200 + PQ) group was still significant from control group regarding cerebral 4-HNE in addition to pulmonary MDA and 4-HNE. Moreover, *Tiron* group showed insignificant difference in MDA and 4-HNE levels compared to control group in both cerebral and pulmonary tissues.

### Assessment of cerebral and pulmonary antioxidants Nrf2 and HO-1 biomarkers

**Fig. 10:**
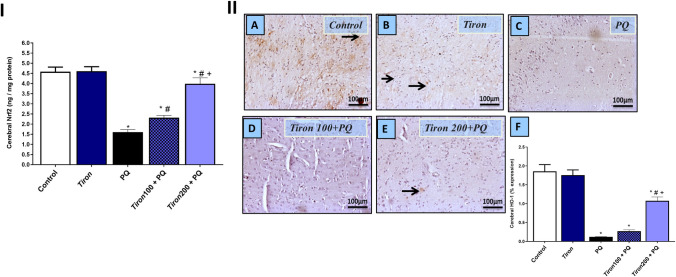
Effect of *Tiron* on cerebral Nrf2 and HO-1 levels. Panel I: Cerebral Nrf2. Panel II: **A**, **B**: sections of brain of control and *Tiron* groups (X: 100, bar = 100 µm); showed showing mild to moderate immunopositive stained neurological cells, **C**: sections of brain of PQ group (X: 100, bar = 100 µm), showed none to few immunopositive stained cells, **D**: sections of brain of (*Tiron* 100 + PQ) group (X: 100, bar = 100 µm); showed few immunostained cells, **E**: sections of brain of (*Tiron* 200 + PQ) group (X: 100, bar = 100 µm); showed mild immunopositive stained cells, **F**: % of cerebral HO-1 expression.. PQ: Paraquat; HO-1: heme-oxygenase1; Nrf2: nuclear factor erythroid 2-related factor 2**.** Data are expressed as mean ± S.D. (*n* = 6). ^*, #, +^ *p* < 0.05 statistically significant difference vs. control, PQ, and (*Tiron* 100 + PQ) groups respectively (One-Way ANOVA followed by Tukey–Kramer multiple comparisons test)

**Fig. 11:**
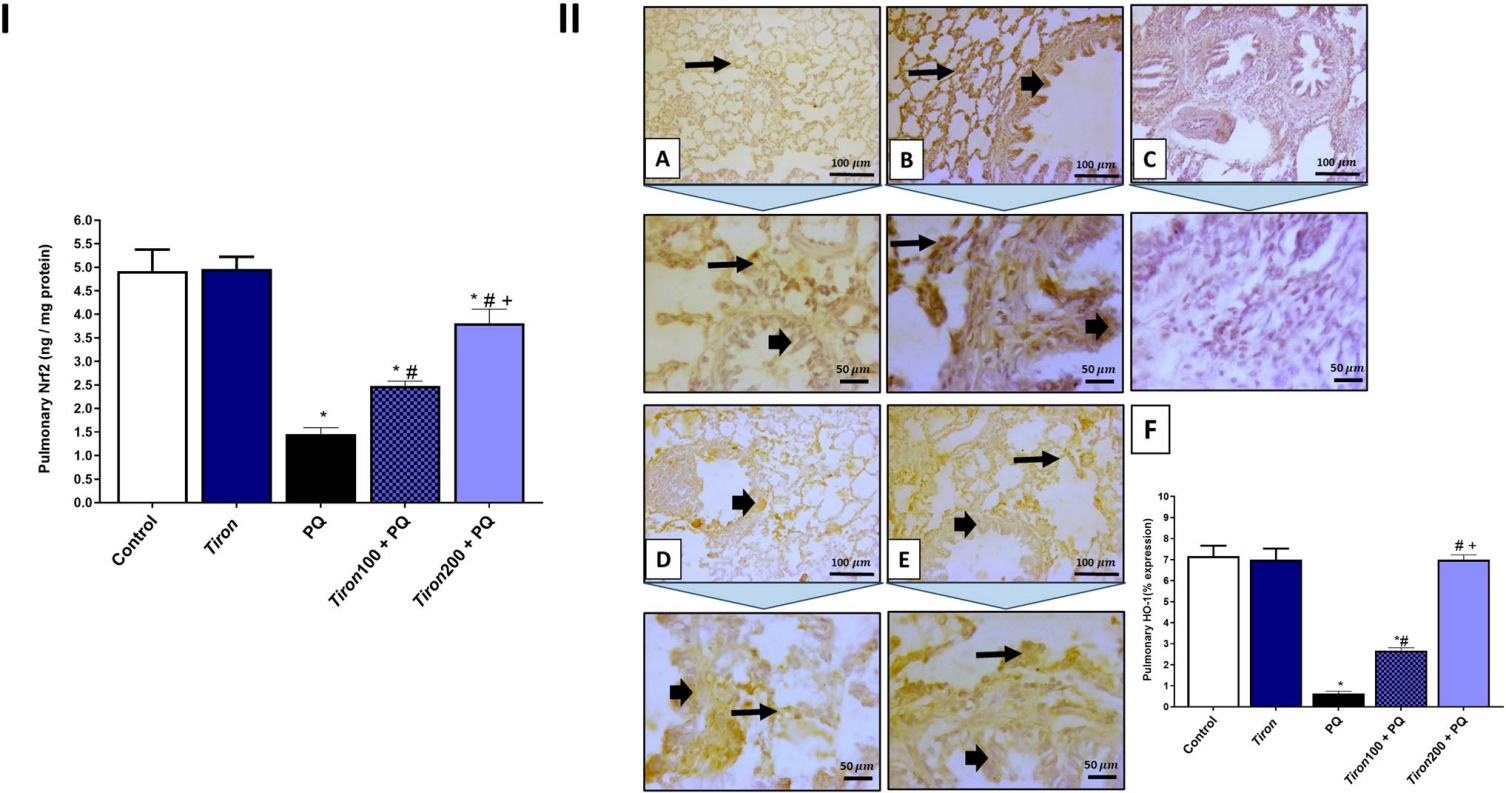
Effect of *Tiron* on pulmonary Nrf2 and HO-1 levels. Panel I: Pulmonary Nrf2. Panel II: Representative IHC of HO1 in pulmonary sections of different treatment groups (low and their corresponding high power view). **A** Control group showing diffuse alveolar and bronchiolar epithelial immunopositive cytoplasmic staining. **B** *Tiron group* showing high immunopositive staining of bronchiolar and alveolar epithelial cells. **C** PQ group showing negative bronchiolar and alveolar epithelial staining. **D** *Tiron* 100 + PQ showing mild to moderate immunopositive stained bronchiolar and alveolar epithelial cells. **E** *Tiron* 200 + PQ showing moderate immunopositive stained alveolar and bronchiolar epithelial cells. Thin arrows = positive alveolar cells, thick arrows = positive bronchiolar cells. Image magnification = 100x = Bar 100 μm, 400x = Bar 50 μm. **F**: % of pulmonary HO-1 expression.. PQ: Paraquat; HO-1: heme-oxygenase1; Nrf2: nuclear factor erythroid 2-related factor 2**.** Data are expressed as mean ± S.D. (*n* = 6). ^*, #, +^ *p* < 0.05 statistically significant difference vs. control, PQ, and (*Tiron* 100 + PQ) groups respectively (One-Way ANOVA followed by Tukey–Kramer multiple comparisons test)

An estimation of cerebral and pulmonary levels of antioxidant transcription factor Nrf2 in cerebral and pulmonary tissues revealed a significant (*p* < 0.05) decrease by 64.93% and 70.35% respectively in PQ group compared to control group (Figs. 10I and 11I). Whereas (*Tiron* 100 + PQ) group achieved a considerable (*p* < 0.05) increase in Nrf2 level in cerebral and pulmonary tissues by 1.44 and 1.7-folds respectively compared to PQ group. Moreover, (*Tiron* 200 + PQ) group showed a significant (*p* < 0.05) increase in Nrf2 level by nearly 2.5 and 2.6-folds respectively compared to PQ group. Hence, (*Tiron* 200 + PQ) group had significantly (*p* < 0.05) better results than (*Tiron* 100 + PQ) group regarding antioxidant Nrf2. Both (*Tiron* 100 + PQ) and (*Tiron* 200 + PQ) groups showed a significant (*p* < 0.05) difference from control group. Moreover, *Tiron* group showed insignificant difference in both cerebral and pulmonary Nrf2 levels compared to control group.

An immunohistochemical assay of cerebral HO-1 (Fig. 10II) showed mild to moderate immunopositive stained neurological cells in both control and *Tiron* groups. In contrast, PQ group showed none to few immunopositive stained cells. In addition, (*Tiron* 100 + PQ) group showed few immunostained cells, and (*Tiron* 200 + PQ) group showed mild immunopositive stained cells. Figure 10II-F showed the alleviating antioxidant effect of *Tiron* groups concerning HO-1 levels in cerebral tissues, as it revealed that PQ toxicity decreased significantly (*p* < 0.05) cerebral HO-1 expression by 93.49% compared to control group, while treatment with 100 and 200 mg/kg of *Tiron* significantly (*p* < 0.05) elevated HO-1 levels by 2.24 and 8.89-folds respectively compared to PQ injected groups but both were still significant (*p* < 0.05) compared to control group. Additionally, it is shown that (*Tiron* 200 + PQ) group had stronger antioxidant activity than (*Tiron* 100 + PQ) group. Moreover, *Tiron* group showed insignificant difference in cerebral HO-1 expression compared to control group.

In addition, an immunohistochemical assay of pulmonary HO-1 (Fig. 11II) showed diffuse positive immunostained cells of alveolar septa in both control and *Tiron* groups. In contrast, PQ group showed none to few immunopositive staining in alveolar septa. In addition, (*Tiron* 100 + PQ) group showed mild immunopositive stained bronchiolar and alveolar epithelial cells, and (*Tiron* 200 + PQ) group showed high, intense immunopositive stained cells in bronchiolar and peribronchiolar alveolar epithelial cells. Figure 11II-F showed the alleviating antioxidant effect of *Tiron* groups concerning HO-1 levels in pulmonary tissues, as it revealed that toxicity with PQ led to an intense decrease in pulmonary HO-1 by 91.24% compared to control group, whereas, treatment with (100 and 200 mg/kg) of *Tiron* significantly (*p* < 0.05) elevated HO-1 levels by 4.26 and 11.14-folds compared to PQ injected group, indicating that (*Tiron* 200 mg/kg) dose had better antioxidant effect than that of (100 mg/kg), and that only (*Tiron* 100 + PQ) group was still significant (*p* < 0.05) compared to control group. Moreover, *Tiron* group showed insignificant difference in pulmonary HO-1 expression compared to control group.

### Assessment of cerebral and pulmonary inflammatory TLR4, NF-κB p65, and IL-1β

**Fig. 12:**
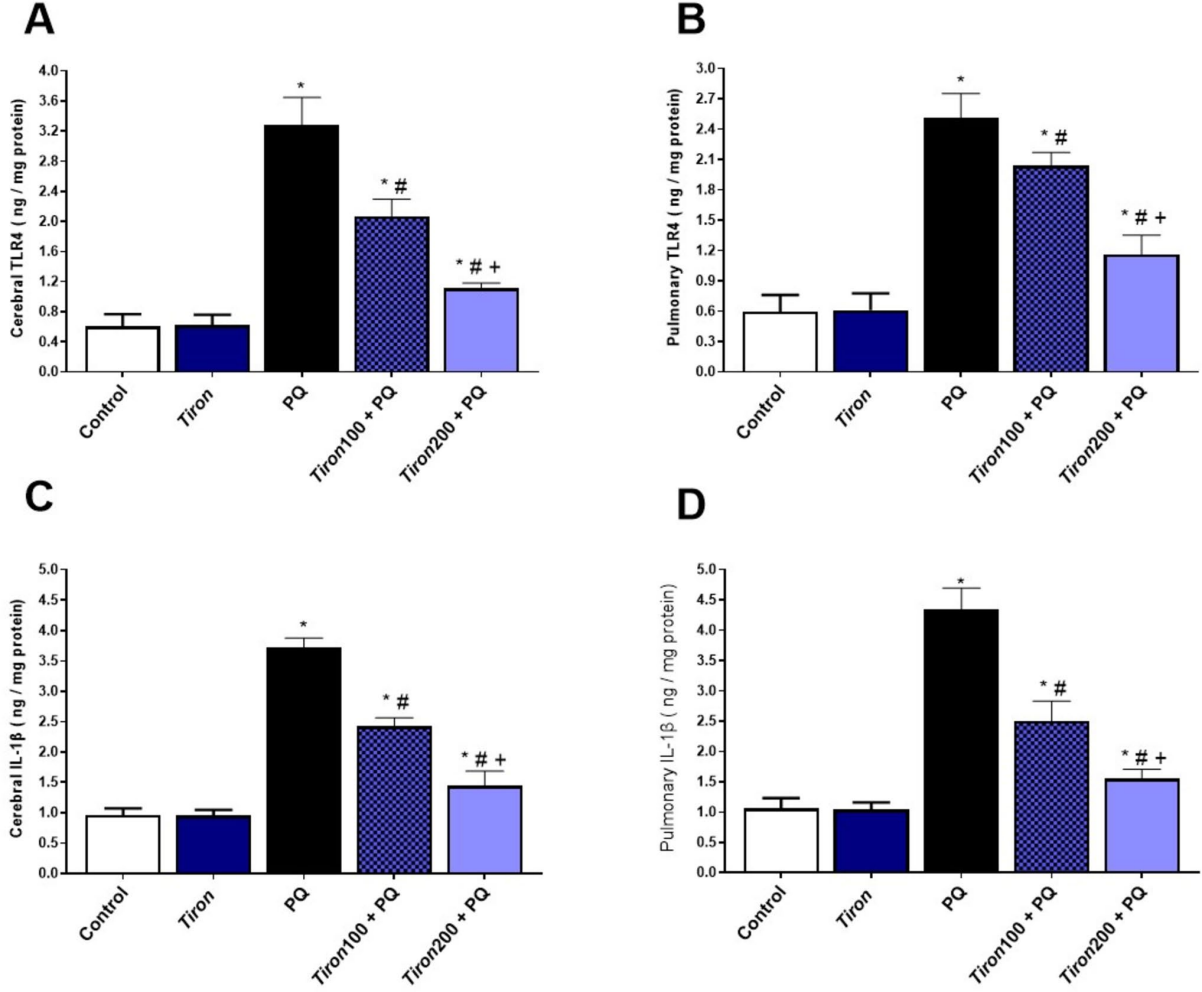
Effect of *Tiron* on cerebral and pulmonary inflammatory TLR4 and IL-1β levels. **A**: cerebral TLR4, **B**: pulmonary TLR4, **C**: cerebral IL-1β, **D**: pulmonary IL-1β. PQ; Paraquat, TLR4; Toll—like receptor 4, IL-1β; interleukin 1beta. Data are expressed as mean ± S.D. (*n* = 6). ^*, #, +^ *p* < 0.05 statistically significant difference vs. control, PQ, and (*Tiron* 100 + PQ) groups respectively (One-Way ANOVA followed by Tukey–Kramer multiple comparisons test)

An estimation of cerebral and pulmonary levels of inflammatory TLR4 and IL-1β revealed a significant (*p* < 0.05) increase in TLR4 and IL-1β in PQ group by nearly 5.4 and 3.9 -folds respectively in cerebral tissues (12A and 12 C), and by 4.2 and 4.1-folds respectively in pulmonary tissues compared to control group (Fig. 12B and D).Whereas, (*Tiron* 100 + PQ) group showed a marked (*p* < 0.05) decrease in the latter markers by 37.12% and 34.82% respectively in cerebral tissues, and by 18.67% and 42.28% in pulmonary tissues in comparison with PQ group. Besides, (*Tiron* 200 + PQ) group showed a significant (*p* < 0.05) decrease in the prementioned biomarkers by 66.08% and 61.11% respectively in cerebral tissues, and by 53.75% and 64.22% respectively in pulmonary tissues when compared to PQ-injected rats, these findings support that (200 mg/kg) of *Tiron* could attenuate inflammation better than (100 mg/kg) of *Tiron*. Both (*Tiron* 100 + PQ) and (*Tiron* 200 + PQ) groups showed a significant (*p* < 0.05) difference from control group. Furthermore, *Tiron* group had no significant difference regarding cerebral and pulmonary TLR4 and IL-1β compared to control group.

**Fig. 13:**
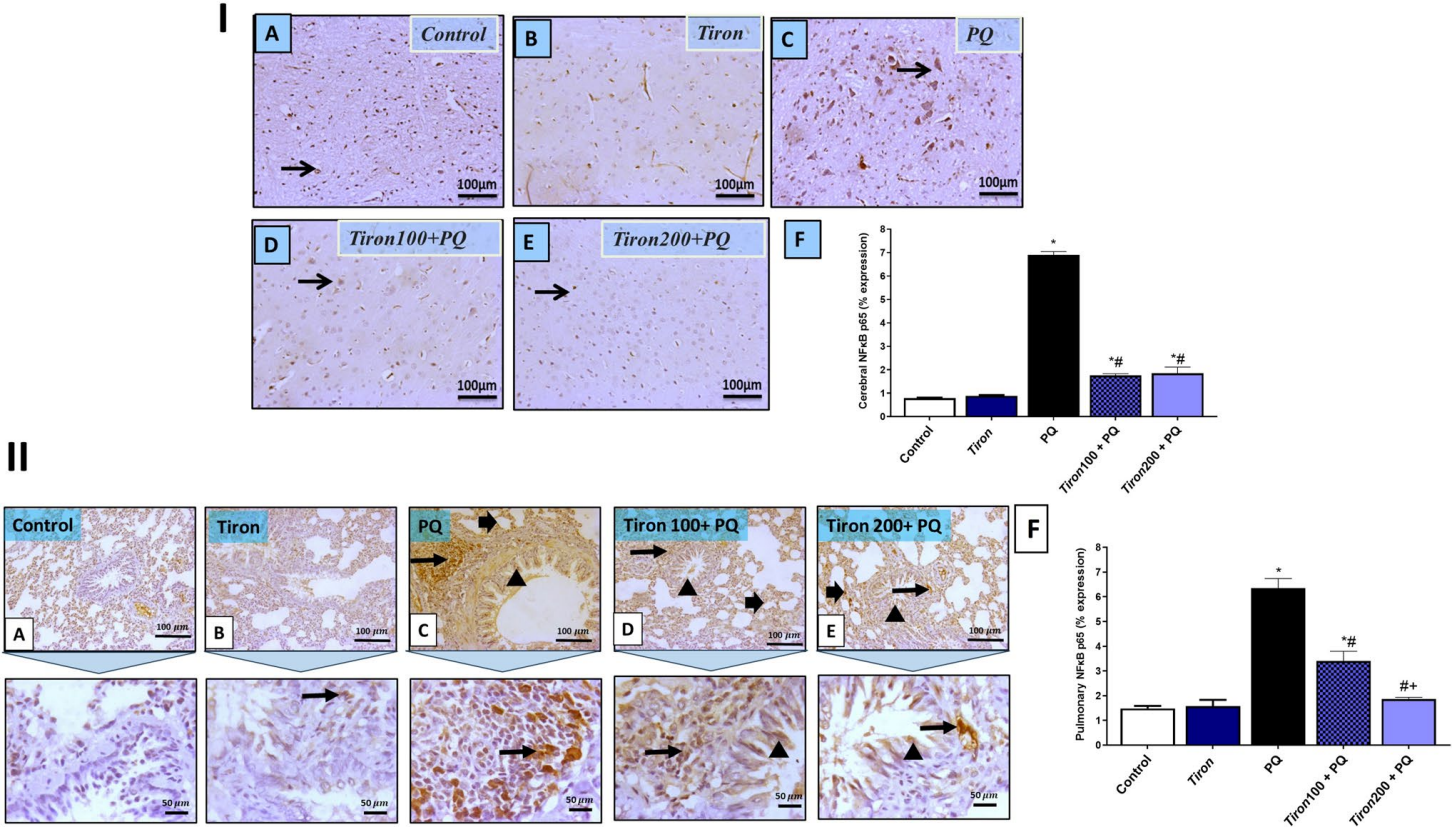
Effect of *Tiron* on cerebral and pulmonary NF-κB p65 levels. Panel I: A,B: sections of brain of control and *Tiron* groups (X: 100, bar = 100 µm); showed none to few faint immunopositive stained neuronal cells, C: sections of brain of PQ group (X: 100, bar = 100 µm, X 400, bar = 50 µm); showed high expression of NF-κB p65 in neuronal cells, D: sections of brain of (*Tiron* 100 + PQ) group (X: 100, bar = 100 µm); showed mild expression in neuronal cells, E: sections of brain of (*Tiron* 200 + PQ) group (X: 100, bar = 100 µm); showed mild immunopositive stained neuronal cells, F: % of cerebral NF-κB p65 expression.. Panel II: Representative IHC of NF-κB p65 expression and localization in pulmonary sections of different treatment groups (low and their corresponding high power view). **A** Control group showing few immunopositive nuclear stained interalveolar cells. **B** *Tiron* group: showing scattered few to mild immunopositive stained bronchiolar, alveolar and interalveolar cells. **C** PQ group: showing high strong immunopositive nuclear stained peribronchiolar expansile inflammatory aggregates with nuclear expression in alveolar and bronchiolar epithelial cells. **D** *Tiron* 100 + PQ group: showing focal peribronchiolar strong immunopositive nuclear stained inflammatory cells. **E** *Tiron* 200 + PQ group: showing scattered few to mild immunopositive stained bronchiolar, alveolar and interalveolar cells. Thin arrows = positive inflammatory or interalveolar resident cells, thick arrows = positive alveolar epithelial cells, arrowheads = positive bronchiolar epithelial cells. Image magnification = 100x = Bar 100 μm, 400x = Bar 50 μm., **F**: % of pulmonary NF-κB p65 expression.. PQ; Paraquat, NF-κB p65; nuclear factor kappa B p65 subunit**.** Data are expressed as mean ± S.D. (*n* = 6). ^*, #, +^ *p* < 0.05 statistically significant difference vs. control, PQ, and (*Tiron* 100 + PQ) groups respectively (One-Way ANOVA followed by Tukey–Kramer multiple comparisons test)

Additionally, an immunohistochemical analysis of cerebral NF-κB p65 (Fig. 13I) revealed none to minimal faint immunopositive stained neuronal cells concerning both control and *Tiron* groups. On the other hand, PQ group showed high expression of NF-κB p65 in neuronal cells, while (*Tiron* 100 + PQ) showed mild expression in neuronal cells. and (*Tiron* 200 + PQ) group showed mild immunopositive stained neuronal cells.

Figure 13I-F describes a comparison of cerebral NF-κB p65 between the different treatment groups showing that percent of expression of cerebral NF-κB p65 was increased by nearly 8.89-folds compared to control group, while treatment with (100 and 200 mg/kg) of *Tiron* decreased significantly (*p* < 0.05) the expression levels by 74.5% and 73.2% respectively compared to PQ group but both groups were still significant (*p* < 0.05) compared to control group. Moreover, *Tiron* group showed insignificant difference in cerebral NF-κB p65 expression compared to control group.

In addition, NF-κB p65 was estimated immunohistochemically in pulmonary tissues (Fig. 13II) to ascertain the inflammatory effect of PQ and revealed minimal immunopositive staining in interalveolar septa in both control and *Tiron* groups. On the other hand, it was shown that PQ group exhibited high, intense expression of NF-κB p65 in peribronchiolar inflammatory aggregates, alveolar, and bronchiolar epithelium, while (*Tiron* 100 + PQ) group showed moderate multifocal immunopositive stained cells in peribronchiolar inflammatory cells with faintly stained bronchiolar and alveolar epithelia cells, and (*Tiron* 200 + PQ) rats showed scattered few positive stained cells of alveolar and bronchiolar epithelial cells.

Figure 13II-F describes a comparison of pulmonary NF-κB p65 between different treatment groups versus control and PQ groups, showing an intense significant increase in percent of pulmonary NF-κB p65 expression by 4.28-folds in PQ injected rats when compared to control group, while treatment with (100 and 200 mg/kg) of *Tiron* decreased significantly (*p* < 0.05) these expression levels by 46.34% and 70.68% respectively compared to PQ group. Moreover, (*Tiron* 100 + PQ) group showed a significant (*p* < 0.05) difference from control group regarding pulmonary NF-κB p65. *Tiron* group showed insignificant difference in cerebral NF-κB p65 expression compared to control group.

## Discussion

PQ is recognized as an extremely lethal herbicide causing multiple organ damage and affect both brain and lung, and its deleterious effects lead to pulmonary fibrosis, acute respiratory distress syndrome, cerebral damage, and memory disorders, through the activation of oxidative stress cascade followed by a highly intensive inflammatory reaction (Daneshvar et al. 2022; Yadav et al. 2024). The brain is highly susceptible to PQ toxicity, as PQ has been extensively discussed for its potential to induce parkinsonism, behavioural changes, and neuroinflammation in different species of rodents (Imam et al. 2024; Atone et al. 2023; Beigoli et al. 2024). Pulmonary tissues are also affected as acute lung injury is considered the leading cause of death following PQ intoxication and previous studies had focused on oxidative stress and inflammation propagated by PQ that extensively damaged pulmonary tissues in different species (Cui et al. 2023; Beigoli et al. 2024).Findings of our study showed that PQ administration resulted in clear histopathological alterations in pulmonary, cerebral tissues and motor dysfunction.

The present study showed a significant deterioration in pulmonary and cerebral oxidant/antioxidant status reflected by decrease of their levels of GPX4, GSH, Nrf2, and HO-1 and increase in their MDA levels. Furthermore, a heightened overall count of leucocytes, along with high infiltration of inflammatory cells in the pulmonary microenvironment in PQ group was noted beside high levels of LDH activity and TP content. PQ exerts a dramatic disturbance in oxidant/antioxidant balance within brain and lungs (Beigoli et al. 2024; Saberi-Hasanabadi et al. 2024). An abundance of free radicals is generated upon exposure to PQ which leads to a catastrophic damage in proteins, lipids, and nucleic acids ultimately causing disturbances in cellular metabolism (Robb et al. 2015). Studies have demonstrated that an excess of free radicals generated by PQ can harm structures within the brain, potentially leading to cell death, thereby increasing the risk of motor dysfunction which was supported by the wire hanging and OFT test (Jenner 2003; Li et al. 2016). Similarly, PQ induced oxidative stress effects on pulmonary tissue were deeply investigated in a previous study of Liu et al. (2022), in which excessive free radicals generated by PQ caused cellular damage via the depletion of NADPH which is a co factor for glutathione reductase enzyme helping in the regeneration of GSH, this depletion leads to the accumulation of oxidized form of glutathione (GSSG) resulting in the formation of protein mixed disulfides contributing to lung toxicity (Liu et al. 2022). As lipid peroxidation is considered the first crucial step in the pathophysiology of PQ intoxication by affecting cell membrane and damaging mitochondria, MDA and 4-HNE are found to be highly elevated in cases of PQ toxicity as a marker of lipid peroxidation, along with a decrease in antioxidants, GPX4, GSH and TAC (Liu et al. 2022), a fact that is consistent with our results upon PQ injection.

*Tiron* has a documented protective effect against oxidative stress as it has the ability to scavenge various radicals (Kim et al. 2006). The study of Oyewole and Birch-Machin (2015) revealed that the small size of *Tiron* makes it easy to enter inside cells and then modify intracellular electron transfer reactions by a free radical scavenging mechanism (Oyewole and Birch-Machin 2015). In the present study, *Tiron* -treated rats showed better motor activity in wire hanging and OFT and showed restored oxidant/antioxidant in cerebral and pulmonary indicating *Tiron* strong antioxidant activity.

Ferroptosis is a newly discovered type of regulated cell death that depends mainly on the imbalance between intracellular lipid ROS generation and degradation (Chen et al. 2021). Reduction of cells antioxidant ability causes iron to generate uncontrolled amounts of ROS triggering ferroptosis, these ROS are dramatically unstable with high energy and prone to sudden energy loss leading to cell death (Yang and Lian 2020). It has been demonstrated that 4-HNE and MDA, an indicators of lipid peroxidation, consequently rate the cellular ferroptosis process (Zhang et al. 2023). It has been known that metals such as iron may play a vital role in various diseases especially conditions related to heavy accumulation of ROS (Mena et al. 2015). Iron ions, which are considered the major inducers of ferroptosis, usually enter cells as ferric ions (Fe^+3^) where they are reduced to ferrous ions (Fe^+2^) via ferric reductase enzyme, the latter ions catalyse Fenton reaction which stimulates ROS production, then these ROS subsequently tend to oxidize nontoxic phospholipids into toxic peroxidised lipids (Stockwell et al. 2017). It has been documented in recent studies that intoxication with PQ could decrease cell viability through the accumulation of free iron along with ferritin degradation (Du et al. 2024). So, agents with ability of iron chelation such as *Tiron* may be beneficial as ROS scavenger as it has been documented that the efficacy of *Tiron* against oxidative stress inducers is attributed to its antioxidant and metal chelation properties (Oyewole and Birch-Machin 2015). In the current study, serum iron was markedly elevated in PQ-injected rats along with abnormally reduced serum ferritin level, elevated pulmonary and cerebral 4-HNE and MDA reflecting the intense accumulation of iron within our studied cerebral and pulmonary tissues indicating high ferroptotic activity, while *Tiron*- treated groups exhibited lower risk of ferroptotic cell death. These results were supported by a previous study of Subburaya et al. (2020) on the efficacy of *Tiron* to suppress ferroptosis in cells of human beings (Subburayan et al. 2020).

GPX4 is considered a central regulator to ferroptosis, as by the use of GSH, GPX4 can convert the cytotoxic lipid peroxides into the corresponding nontoxic alcohol, losing its peroxide activity, hence supressing cell death and that what was found in the current study, as GPX4 and GSH were both downregulated in cerebral and pulmonary tissues of PQ-injected group, which match with the previous results deduced by Yang et al. (2023), and Ijaz et al. (2024) (Yang et al. 2023; Ijaz et al. 2024). On the other hand, *Tiron*-treated groups showed higher levels of cerebral and pulmonary GPX4 and GSH, which indicates that *Tiron* could downregulate ferroptotic activity because of its ability in scavenging free radical and iron chelation.

Nrf2 is considered a transcription factor that controls cellular antioxidant response, as it regulates the transcription and regeneration of GSH as well as antioxidant enzymes HO-1, GPX4, GST, superoxide dismutase, and catalase, so as previously demonstrated by Liu et al. (2022), and Ijaz et al. (2024), Nrf2 has a critical role in the generation of NADPH and detoxification of ROS and a subsequent protection against cytotoxicity, mitochondrial dysfunction, and ferroptosis (Liu et al. 2022; Ijaz et al. 2024). Under normal conditions, Nrf2 is located in the cytoplasm, where it is sequestered by Keap1, which is considered an adaptor protein that undergoes continuous ubiquitylation and degradation of Nrf2 (Tonelli et al. 2018). While, upon heavy generation of ROS, Keap1’s dissociation from CUL-E3 ligase is activated, followed by alteration of cysteine residues of Keap1, followed by the translocation of Nrf2 into the nucleus (Yang et al. 2023; Liu et al. 2022), leading to the malfunction of the antioxidant defense mechanism and disturbance in the homeostasis of the antioxidant protein, Nrf2 (Ikram et al. 2019; Woodburn et al. 2021). In the present study there was a marked downregulation in Nrf2 in PQ groups in both cerebral and pulmonary tissues compared to the control group, an effect that was reversed by *Tiron* administration. As discussed before, the most antioxidant gene that Nrf2 activates is HO-1. The current study demonstrated marked downregulation of HO-1 upon PQ intoxication compared to the control group, while *Tiron* administration significantly mitigated the downregulation precipitated by PQ in both cerebral and pulmonary tissues, which reflects its potent antioxidant activity. The relationship between oxidative stress and inflammation has been previously affirmed, as the production of heavy amounts of ROS leads to the activation of various inflammatory responses as a defense mechanism (Popa-Wagner et al. 2013; Hussain et al. 2016). Among these activated inflammatory pathways is the TLR4/NF-κB pathway, in which TLRs, which are a class of proteins that can recognize damage- associated molecular patterns (DAMPs), are activated upon heavy generation of ROS (Liu et al. 2022). Moreover, the activation of the active form of TLR4 leads to further upregulation of NF-κB p65, which subsequently activates DNA response elements, including like IL- 6 and IL-1β (Akhigbe et al. 2024). Furthermore, it is well documented that PQ intoxication leads to activation of the TLR4/NF-κB pathway in both cerebral and pulmonary tissues, as mentioned by Liu et al. (2022), and Atone et al. (2023) (Atone et al. 2023; Liu et al. 2022). Similarly, in our study, the PQ group had the highest levels of TLR4, NF-κB p65, and IL-1β, while as a strong anti-inflammatory, *Tiron* mitigated the severe inflammation induced by PQ with a marked decrease in the latter markers in both cerebral and pulmonary tissues. Additionally, the present study is considered the first study to test the role of *Tiron* in targeting inflammation precipitated by the TLR4 pathway in cerebral and pulmonary tissues.

*Tiron* shows promising translational potential as an antidote due to its dual ability to chelate iron and scavenge ROS, mechanisms that directly target PQ-induced oxidative and inflammatory injury. These properties suggest that *Tiron* could be beneficial in clinical settings as a supportive therapeutic agent and its role in oxidative stress modulation, nanotechnology applications, and targeted molecular pathways in inflammation and cancer requires additional broader research and investigations (Aglan et al. 2024; Abdel-Salam et al. 2012; Melegy et al. 2024; Abd-Rabou et al. 2025).

The higher dose of *Tiron* was more effective for some parameters (e.g., ferritin, Nrf2) but not others (e.g., cerebral NF-κB p65, where 100 and 200 mg/kg had similar effects). The differential response to *Tiron* dosing suggested that its therapeutic effects were dose-dependent but pathway-specific. For parameters such as ferritin and Nrf2, the higher dose (200 mg/kg) produced greater improvement, likely because these targets were closely linked to iron chelation capacity and antioxidant enhancement. Increasing the dose may had provided a stronger reduction in free iron and ROS, thereby promoting a more pronounced activation of the Nrf2–HO-1 pathway. In these parameters, the effect appears linear or dose-responsive, where more Tiron leads to greater impact. In contrast, cerebral NF-κB p65 did not show a markedly higher response at 200 mg/kg compared to 100 mg/kg, suggesting a threshold or saturation effect. Once NF-κB signalling is sufficiently suppressed at the moderate dose, additional Tiron may not yield further inhibition. This plateau may indicate that the NF-κB pathway becomes maximally inhibited at lower concentrations, Tiron’s penetration across the blood–brain barrier may limit additional benefit at higher doses and distinct sensitivity of signalling pathways causes some to respond strongly to dose escalation (Nrf2), while others reach an efficacy ceiling earlier (NF-κB).

However, several limitations remain: the current findings are based primarily on pre- or co-treatment models that do not fully reflect real-world intoxication scenarios, and variability in dose responsiveness across pathways (e.g., Nrf2 vs. NF-κB) raises uncertainty about the optimal therapeutic window. Future studies should therefore focus on evaluating *Tiron* in true post-exposure therapeutic models, optimizing dosing strategies, assessing pharmacokinetics and tissue distribution, and confirming safety and efficacy in more clinically relevant designs. In addition, future studies should incorporate both sexes to elucidate sex-dependent mechanisms and optimize therapeutic strategies accordingly.

## Conclusion

**Fig. 14:**
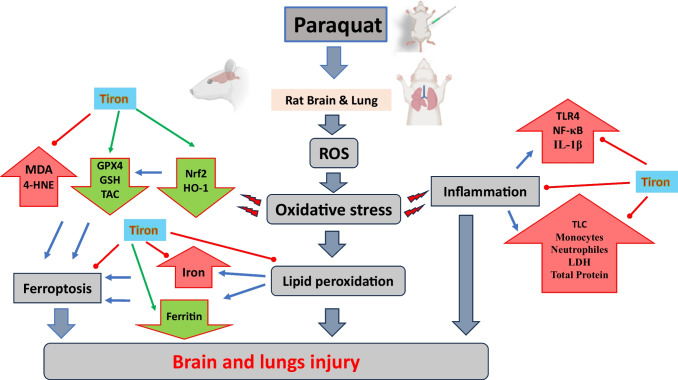
Schematic diagram of possible mechanisms by which *Tiron* may attenuate cerebral and pulmonary injury precipitated by PQ. PQ: Paraquat, OFT; Open field test, BAL fluid; Bronchoalveolar lavage fluid, LDH: Lactate dehydrogenase, TLC: Total leucocytes count, GPX4; Glutathione peroxidase 4, GSH: Reduced glutathione, TAC: Total antioxidant capacity, MDA: Malonaldehyde, 4-HNE; 4-hydroxy-2-nonenal, Nrf2; Nuclear factor erythroid 2–related factor 2, HO-1: heme-oxygenase 1, TLR4: toll like receptor 4, NF-κB p65; Nuclear factor kappa B p65 subunit, IL-1β: Interleukin 1β

As shown in Fig. 14, *Tiron* attenuated the deleterious cerebral and pulmonary injury induced by PQ through mitigating oxidative stress, ferroptosis, and inflammation. This was evidenced by the downregulation of the TLR4/NF-κB inflammatory pathway, upregulation of the Nrf2/HO-1 antioxidant pathway, and suppression of lipid peroxidation and iron accumulation within cerebral and pulmonary tissues. Together, these mechanistic findings support the observed statistical differences are accompanied by coherent biological responses with physiological relevance, rather than numerical changes alone.

## Data Availability

All source data for this work (or generated in this study) are available upon reasonable request.
